# How the brain copes with bad times

**DOI:** 10.1371/journal.pbio.3003710

**Published:** 2026-04-03

**Authors:** Joshua L. Gowin, Colleen M. Sheller

**Affiliations:** Department of Radiology, University of Colorado Anschutz Medical Campus, Aurora, Colorado, United States of America

## Abstract

How does the brain represent and manipulate negative emotional experiences? This Primer explores a new study in PLOS Biology that describes how different brain systems correspond to emotion regulation and suggests a key dimension that explains how we feel.

## Teaser

A unifying framework of the brain’s representation and manipulation of negative emotional experiences has been elusive. A new study in *PLOS Biology* describes how different brain systems correspond to emotion regulation and suggests a key dimension that explains how we feel.

Heartache, grief, cuts, and bruises can be put in the same category as death and taxes: humans are certain to encounter them. What is uncertain is the extent that an individual will suffer when they experience one of these tribulations. Our choices when experiencing distress can influence the emotions we have and how they are expressed, and this process is called emotion regulation [1]. While psychological investigation has shown that regulation strategies like rumination and avoidance tend to be associated with greater suffering, problem-solving and mindful acceptance can help reduce the likelihood that challenging circumstances will precipitate episodes of depression [2]. Brain scans that are collected while research participants view unpleasant images or videos and try to reduce their negative experience by thinking about the stimuli differently have found that some regions, such as the dorsomedial prefrontal cortex, are more active during emotion regulation [3]. Activation of such regions during emotion regulation may contribute to inhibition and cognitive control [4]. However, a unifying framework of the brain’s role in emotion regulation has been unclear. A recent study in *PLOS Biology* by Wang and colleagues [5] attempted to describe the brain activation during emotion regulation using a whole-brain approach that considered broad classifications of brain-function relationships to characterize what the brain does when it regulates emotion, and how that processing relates to an individual’s emotional experience. Could a comprehensive metric of brain function provide information beyond what individual regions indicate about how the brain regulates emotions?

**Fig 1:**
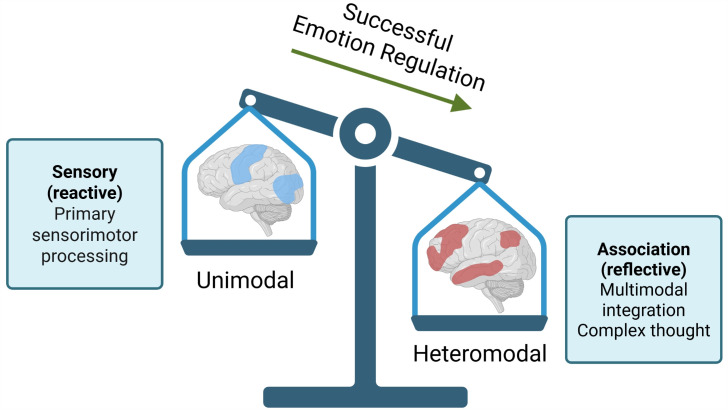
Shifting of sensory-association gradients in emotion regulation. Wang and colleagues found that emotion regulation during an fMRI paradigm correlated with a shift toward employing associative brain regions over sensory regions. This finding helps uncover how the brain must globally shift its capacities to higher-level processing for cognitive reappraisal, rather than solely employing spatially and functionally disparate regions. Created in BioRender. Sheller, C. (2026) https://BioRender.com/3scouv2.

In this study, instead of focusing on individual brain regions, Wang and colleagues used brain-states, which better capture the dynamic nature of how regions across the brain work together. These brain states were generated from hundreds of functional MRI scans done on people at rest, from a large dataset known as the Human Connectome Project [6,7]. The brain-states that Wang and colleagues examined indicated five different dimensions, or gradients: (1) sensory regions versus association cortex, (2) visual versus sensorimotor, (3) default-mode network versus frontoparietal systems, (4) ventral versus dorsal attention networks, and (5) visual cortex versus ventral attention [7]. They compared the brain states generated from the Human Connectome Project to fMRI data collected on people completing an emotion regulation paradigm while in the scanner [5]. Of these dimensions, they found that emotion regulation showed the strongest association with dimension 1, where more successful regulation corresponded to greater shifts toward association processing brain regions, and away from regions primarily associated with sensory processing (Fig 1). For example, a primary sensory region is the visual cortex, and an associative region is the anterior cingulate cortex, and individuals who showed greater shifts from visual to anterior cingulate cortex activation patterns during emotion regulation reported feeling better after viewing the disturbing picture. This suggests that the regions involved in successful regulation are ones that help an individual incorporate multiple pieces of information. Feeling better might mean using the brain to understand a bad circumstance from a broader perspective.

One of the challenges of brain imaging studies is that common analytic procedures produce a list of regions that exceed a statistical threshold, but the regions can be far apart and be associated with vastly different functions. Integrating the meaning of a long list of brain regions that activate can be challenging, and sometimes the result is that a few trees are described, but the forest is missed. The advantage of the brain-state analysis used in the present study is that it summarizes dynamic activation patterns across the brain into a coherent network of functions that can indicate mental performance. For example, previous work has shown that the transition from a more sensory to an association dimension evolves during development and corresponds to the development of more abstract thinking abilities as individuals mature [8]. While previous studies have shown which brain regions are involved in emotion regulation, this study provided a common theme to what the brain regions involved in regulation do. The authors described the forest. This might make it easier for future studies to compare across groups with different emotion regulation abilities, such as individuals with and without psychiatric disorders [9].

While this study represents a notable advance, there were some limitations. The imaging task only used pictures to induce negative emotion states, so it is unclear if successful emotion regulation in other contexts will be associated with a shift along gradient 1. For example, some studies ask individuals to reconsider stressful events from their life, or to watch disturbing movie scenes, and these may have different neural patterns. Similarly, the emotion regulation strategy in this task was cognitive reappraisal, which requires thinking differently about a circumstance with the goal of changing feelings [10]. Other types of emotion regulation, such as acceptance, may have different neural associations. Although the study identified a relationship between neural activation patterns and self-reported emotion regulation success, there was not a relationship with a questionnaire measure of cognitive reappraisal, so it will be important to determine how this neural pattern is reflected in daily life in participants. The study did find a significant association between greater shifts towards association networks during cognitive reappraisal and lower negative affect in daily life, measured by smartphone-based experience sampling in a subset of the sample. This suggests the results may be applicable outside of purely a laboratory setting [5]. Lastly, the participants in the study largely came from Austria, Germany, Canada, and the United States, so it would be important to determine if these results generalize to individuals from other regions.

This work provides a foundation for future investigations into the brain’s role in emotion regulation. Future studies should characterize whether the gradient mapping in the context of emotion regulation can indicate psychopathological states, such as major depressive disorder. If this gradient approach to emotion regulation can distinguish between healthy adults and adults with depression, it could help map neural dysfunction associated with the disorder and indicate when a person improves following treatment. For treatment, it may be important to know if people who show less gradient shifting are more likely to benefit from therapy targeting emotion regulation, because they have more to gain, or alternatively if people who show greater shifts are more susceptible to improvement following treatment targeting emotion regulation. This would be an exciting development.
